# Review of the interactions between G6PD deficiency and primaquine therapy for malaria control and elimination

**DOI:** 10.1186/1475-2875-13-S1-O11

**Published:** 2014-09-22

**Authors:** Rosalind Howes

**Affiliations:** 1 University of Oxford, UK

## 

Primaquine is an essential drug for malaria control and elimination by being the only drug active against the liver-reservoir of *Plasmodium vivax* infections and also the only drug against the mature sexual stages of *Plasmodium falciparum* which are responsible for onward transmission to mosquitoes. Nevertheless, uncertainty associated with this drug’s safety results in under-use due to difficulties with diagnosing glucose-6-phosphate dehydrogenase deficiency (G6PDd) in point-of-care settings. This common genetic disorder makes patients susceptible to potentially severe, and occasionally even fatal, primaquine-induced haemolysis.

Policy shifts towards malaria elimination in recent years have led to a surge of clinical trials and reviews of historical data to promote safe primaquine use. This talk will examine evidence of the drug’s use and outcomes in geographic regions with distinct epidemiological characteristics. For each setting, the parasite epidemiology, the population’s G6PDd characteristics, and the current primaquine policies will be discussed. The quality and completeness of the parasitological, G6PDd and clinical evidence-bases that inform primaquine policy is examined; the importance of context-specific evaluations of primaquine use is emphasized.

